# Cultural Perspectives on Sodium Intake Reduction Among Nigerians in the US: An Application of the PEN-3 Model

**DOI:** 10.5888/pcd18.210159

**Published:** 2021-08-26

**Authors:** Oluwaseun B. Ikuomola, Collins O. Airhihenbuwa

**Affiliations:** 1 School of Public Health, Georgia State University, Atlanta Georgia; 2 Health Policy and Behavioral Sciences, School of Public Health, Georgia State University, Atlanta Georgia

## Introduction

Noncommunicable diseases (NCDs) are a growing source of concern and a challenge to public health. Before the surge of deaths from COVID-19, these diseases accounted for 7 of 10 deaths globally, exceeding deaths from all infectious diseases combined (1). The Global Burden of Disease study of 2017 showed that the leading contributors to NCD-related morbidity and mortality were cardiovascular diseases such as high blood pressure, stroke, coronary heart disease, and heart failure (2). Of these, high blood pressure is one of the most significant (3).

High blood pressure develops from complex and interrelated factors, including those related to modifiable behavior and lifestyle (4). A key modifiable risk factor for NCDS is high sodium intake (4). The World Health Organization (WHO) recommends a daily sodium consumption of less than 2 grams (or <5 g salt/d). If adhered to, this recommendation would prevent an estimated 2.5 million deaths from NCDs worldwide (3). Dietary sodium comes from 3 distinct sources: packaged foods, food prepared outside the home (eg, restaurants), and additional discretionary sources (adding salt to foods prepared at home during cooking or eating) (4). However, sources of dietary sodium intake differ among populations. An understanding of cultural variation in diet and dietary practices that affect sodium intake has important implications for public health practice (4).

## Dietary Sodium Intake Among Nigerians in the US

The source of dietary sodium intake for most Americans is packaged, processed, store-bought, and restaurant foods; hence, these are the focus of population-level sodium reduction interventions (5). However, the source of dietary sodium among Nigerians in the US differs from that of Americans. Nigerians maintain their traditional diet and dietary practices while navigating new cultural spaces and seldom consume packaged or processed foods (6).

Despite the presence of multiple ethnicities in Nigeria, the typical traditional diet across ethnic groups comprises mainly the pairing of carbohydrate-based meals with soup or sauce (7) prepared by adding both salt and bouillon seasoning to enhance the flavor (8). This practice of using both salt and bouillon seasoning in meal preparation (8) has also been observed in metropolitan Atlanta among a sample of Nigerian women, the primary food-preparers for their households (9). This indicates that this salt intake practice persists among Nigerians in the US. Considering that the bouillon seasonings used during food preparation already contain salt, hydrogenated oil, disodium inosinate, monosodium glutamate, and other additives (8), we believe that their combination with salt in meals likely increases sodium consumption above WHO-recommended levels (3). Moreover, monosodium glutamate has been implicated in increased risk of high blood pressure (10,11). Therefore, Nigerians in the US are at risk of high blood pressure because they maintain their traditional meal preparation practices (6) by combining salt with bouillon, which they can access in African stores that sell these seasonings (8).

## Strategies for Reducing Dietary Sodium Intake

WHO’s population-level strategies for sodium intake reduction, which have been adopted in several national, state, and community programs in the US (12), fall into 3 categories: 1) product reformulation of industrially produced foods, 2) consumer awareness and education, and 3) national policies that target standards for food manufacturers and providers (3,4,13). The product reformulation strategy supports sodium intake reduction through collaboration with food producers and distributors to control sodium content of packaged foods and out-of-home or restaurant foods. The consumer awareness and education strategy focuses on individual knowledge, attitudes, and behaviors regarding salt intake. Finally, national policy changes are aimed at food producers and sellers by lowering salt content targets and improving standards while ensuring the affordability and availability of healthy food choices for consumers (13).

Because the source of dietary sodium for most Americans (5) differs from that of Nigerians (6,8), we believe that strategies for reducing sodium intake for the general American population may not be appropriate for Nigerians who live in the US. Interventions directed at US Nigerians should target discretionary sources of sodium and employ the consumer awareness and education strategy in a more culture-centered approach. Interventions should account for certain unique cultural and traditional dietary practices in communication messages to engage Nigerian women because of their role as primary household dietary decision-maker (14).

## Cultural Approach For Dietary Sodium Intake Reduction: The Pen-3 Model For Future Direction

To ensure that interventions to reduce sodium intake based on the consumer awareness and education strategy are culturally centered for Nigerians in the US, public health programs and campaigns should be planned, designed, and implemented by using a cultural model such as PEN-3. Unlike other models or frameworks, the PEN-3 model, which was developed in 1989 (15), centralizes culture as an integral aspect of health behaviors. These behaviors are to be considered at the core of communicating health risks when developing, implementing, and evaluating health interventions (15,16).

The PEN-3 model comprises 3 domains — cultural identity, relationships and expectations, and cultural empowerment. Each of the 3 domains has 3 constructs that form the acronym PEN: 1) person, extended family, and neighborhood (cultural identity domain); 2) perceptions, enablers, and nurturers (relationships and expectations domain); and 3) positive, existential, and negative (cultural empowerment domain). The domains are described in detail elsewhere (8). When designing a culture-sensitive intervention at the community level to reduce salt intake, the PEN-3 model can be applied to address the combined use of salt and bouillon and explore alternative seasonings that contain no monosodium glutamate and very little salt and are therefore healthier. This process can be completed in 2 phases: the assessment phase (relationships and expectations domain, cultural empowerment domain) and the intervention phase (cultural identity domain to identify the point of entry of the intervention) (17). The PEN-3 model has been used in health promotion and education programs to assess nutrition-related attitudes and identify channels for nutrition education among African Americans (18), to explore barriers and facilitators to a healthy diet for parents of young children (19), and to explore perceptions of dietary behavior and habits among US transnational African migrants and Congolese immigrants living in the US (20,21).

In addition to applying the PEN-3 model to planning, designing, and implementing interventions, future efforts should also leverage the potential role that African food stores could play in increasing their inventories of healthier seasoning alternatives. Also, clinicians, particularly those in primary care, could ask their Nigerian patients about their salt intake, thus bringing attention to the matter. For example, clinicians can ask about their diets (6), explaining that high sodium intake predisposes them to high blood pressure or makes blood pressure control more difficult for those with hypertension. Engaging both food markets and clinicians can make Nigerian women aware of the relationship between sodium intake and health and allow them to maintain their cultural preferences for taste and flavor while reducing the risk of chronic disease.
